# miRNA Signatures as Predictors of Therapy Response in Castration-Resistant Prostate Cancer: Insights from Clinical Liquid Biopsies and 3D Culture Models

**DOI:** 10.3390/genes16020180

**Published:** 2025-02-01

**Authors:** Jonathan Puente-Rivera, Stephanie I. Nuñez-Olvera, Verónica Fernández-Sánchez, Monica Alethia Cureño-Díaz, Erika Gómez-Zamora, Estibeyesbo Said Plascencia-Nieto, Elisa Elvira Figueroa-Angulo, María Elizbeth Alvarez-Sánchez

**Affiliations:** 1 Laboratorio de Patogénesis Celular y Molecular Humana y Veterinaria, Posgrado en Ciencias Genómicas, Universidad Autónoma de la Ciudad de México (UACM), San Lorenzo 290, Col. Del Valle, Mexico City 03100, Mexico; jo_puenter@hotmail.com (J.P.-R.);; 2 División de Investigación, Hospital Juárez De México, Mexico City 07760, Mexico; 3 Departamento de Biología Celular y Fisiología, Instituto de Investigaciones Biomédicas, Universidad Nacional Autónoma de México (UNAM), Mexico City 04510, Mexico; 4 Facultad de Estudios Superiores Iztacala, Universidad Nacional Autónoma de México (UNAM), Tlalnepantla de Baz 54090, Mexico; 5 Dirección de Investigación y Enseñanza, Hospital Juárez de Mexico, Mexico City 07760, Mexico; 6 Departamento de Salud Pública, Facultad de Medicina, Circuito Interior, Ciudad Universitaria UNAM, Mexico City 04510, Mexico; 7 Dirección Médica, Hospital Juárez de México, Mexico City 07769, Mexico; 8 Sección de Estudios de Posgrado e Investigación, Escuela Superior de Medicina, Instituto Politécnico Nacional, Mexico City 11340, Mexico

**Keywords:** prostate cancer, miRNAs, responder patients, CRPC

## Abstract

Background/Objectives: Prostate cancer (PCa) patients who do not respond to androgen deprivation therapy (ADT), referred to as castration-resistant prostate cancer (CRPC), remain a clinical challenge due to confirm the aggressive nature of CRPC and its resistance to conventional therapies. This study aims to investigate the potential of microRNAs (miRNAs) as biomarkers for predicting therapeutic response in CRPC patients. Methods: We performed miRNA and mRNA expression analyses using publicly available datasets and applied 3D cell culture models to replicate more physiologically relevant tumor conditions. Genetic analysis techniques were employed on publicly available data, and expression profiles from 3D cell culture models were examined. Results: Eighteen miRNAs with differential expression were identified between patients who responded favorably to abiraterone therapy (responders) and those with advanced CRPC (non-responders). Specifically, miRNAs such as hsa-miR-152-3p and hsa-miR-34a-3p were found to be associated with critical pathways, including TGF-β signaling and P53, which are linked to therapeutic resistance. Several miRNAs were identified as potential predictors of treatment efficacy, including therapies like abiraterone. Conclusions: These results indicate that miRNAs could serve as non-invasive biomarkers for predicting therapeutic outcomes, facilitating a more personalized approach to CRPC treatment. This study provides a novel perspective on treatment strategies for CRPC, emphasizing the role of miRNAs in improving therapeutic precision and efficacy in this complex disease.

## 1. Introduction

Prostate cancer (PCa) remains one of the most prevalent and deadly malignancies affecting men worldwide, posing a significant burden on global health systems [1,2]. Recent global cancer statistics highlight the magnitude of this issue, with approximately 4 million new cases diagnosed annually and over 375,000 deaths reported in 2020 alone [3]. Despite considerable advancements in diagnostic tools and therapeutic strategies, the clinical management of PCa remains a significant challenge due to the disease’s inherent heterogeneity and the inevitable development of resistance to standard treatments, such as androgen deprivation therapy (ADT) [4,5]. ADT remains the foundation for managing advanced PCA, including castration-resistant PCa (CRPC). Current guidelines recommend combining ADT with androgen receptor (AR) pathway inhibitors (ARPIs) like enzalutamide, apalutamide, or abiraterone acetate for metastatic hormone-sensitive PCa (mHSPC) [6], non-metastatic CRPC (nmCRPC) with rapid PSA doubling times, and metastatic CRPC (mCRPC); docetaxel-based chemotherapy is a standard option, especially after ARPI progression [7]. Genetic testing for DNA repair deficiencies is advised to guide the use of PARP inhibitors [8], and emerging therapies, such as the combination of talazoparib with enzalutamide, show promise for improving overall survival [8]. These strategies emphasize personalized treatment approaches informed by clinical and genetic factors. This heterogeneity manifests at the molecular level through diverse genetic and phenotypic profiles, including the luminal and basal subtypes, which significantly influence treatment responses and resistance mechanisms [9,10,11]. Thus, identifying reliable biomarkers for early detection, accurate prognostication, and therapeutic response prediction is imperative to enhance clinical outcomes and tailor patient care more effectively [12,13,14].

MicroRNAs (miRNAs) have emerged as potential biomarkers due to their pivotal role in regulating gene expression and their detectable presence in various biological fluids, including serum, reflecting their dynamic changes in tumor pathophysiology [15,16]. These small, non-coding RNAs are key modulators of critical cellular processes such as proliferation, apoptosis, and even metastasis events through their ability to target multiple mRNAs simultaneously [17,18,19]. Recent studies have highlighted circulating miRNAs as important contributors to the progression of PCa and CRPC, making them strong candidates for patient stratification and for monitoring therapeutic responses [20,21]. Notably, miRNAs such as miR-21, miR-141, and miR-375 have been implicated in the progression of PCa and the development of resistance to therapies [22,23,24]. Furthermore, the downregulation of tumor-suppressive miRNAs, including miR-4287, an miRNA associated with more advanced stages of the disease [25]. Collectively, these findings emphasize the potential of miRNAs to enable personalized therapeutic strategies tailored to individual molecular profiles. The use of miRNAs as biomarkers is further underscored by their detectability in non-invasive liquid biopsies, such as blood and urine samples, which offer real-time insights into the molecular dynamics of PCa [14,26,27]. Compared to traditional tissue biopsies, liquid biopsies provide significant advantages, including the ability to perform longitudinal monitoring of disease progression and therapeutic responses [28].

However, a comprehensive understanding of miRNAs’ roles as biomarkers necessitates experimental models that accurately capture the complexity of PCa biology. In this context, three-dimensional (3D) cultures, particularly organotypic cultures derived from the PC3 PCa cell line created by growing the cell line within an extracellular matrix (ECM) that serves as a platform for the development of 3D structures and modulating cancer hallmarks like tumorigenesis [29], have emerged as robust tools for studying cellular interactions within a more physiologically relevant environment compared to two-dimensional (2D) monolayers [30,31,32] and are considered to more accurately replicate human tumors compared to cells grown as 2D monolayers on plastic surfaces [33]. These models facilitate three-dimensional cell–cell interactions, the development of representative tumor microenvironments, and a more precise evaluation of therapeutic responses compared to traditional two-dimensional cultures [34,35,36]. In 3D cell culture techniques, the cellular environment can be adjusted to mimic in vivo conditions, providing more reliable insights into cell-to-cell interactions, tumor morphology and molecular characteristics, drug development, metabolic analysis, and stem cell research. Additionally, patient-derived organoids offer an even more personalized platform for studying PCa, as they effectively replicate the molecular and phenotypic diversity observed in clinical cases [37].

Recent advancements in 3D cell culture techniques have led to the development of sophisticated models that replicate the tumor microenvironment. These models have proven instrumental in studying critical biological aspects of tumors, including cell–cell interactions, drug penetration, and mechanisms of resistance [38,39]. Furthermore, 3D cell cultures offer an invaluable platform for evaluating the efficacy of novel therapeutic agents and gaining deeper insights into the molecular mechanisms underlying therapy resistance [40,41,42]. The incorporation of multicellular 3D models—comprising not only cancer cells but also key components of the tumoral microenvironment, including fibroblasts and certain immune cells—has further advanced our understanding of how these interactions contribute to therapeutic resistance [34].

**Figure 1:**
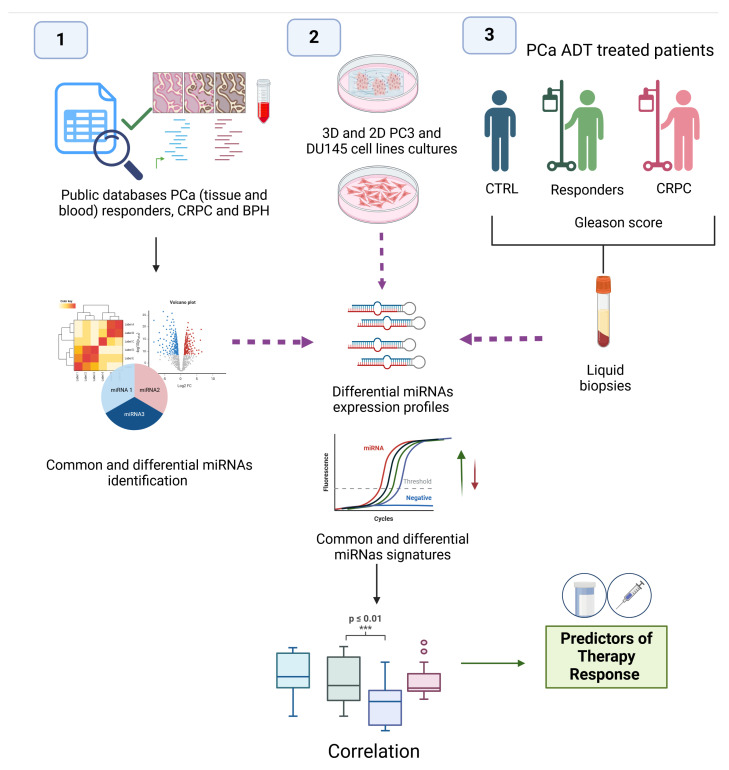
Study workflow for identifying miRNA predictors of therapy response in PCa. Diagram workflow illustrates the multi-step approach used in this study. (1) Data integration and analysis were performed using public PCa databases, including tissue and blood samples from responders, CRPC patients, and BPH cases, to identify common and differential miRNA profiles. (2) PC3 and DU145 PCa cell lines were cultured in both 3D and 2D models to analyze differential miRNA expression profiles and validate their roles in therapeutic resistance. (3) Liquid biopsy samples from ADT-treated PCa patients, categorized as responders, CRPC patients, and CTRL, were analyzed to correlate Gleason scores and miRNA expression. Differential miRNA signatures were correlated with therapy response, generating potential biomarkers to predict therapeutic outcomes in PCa. Purple dotted arrows represent the convergence of in silico data mining, experimental validation, and patient-derived samples. Upward green arrows indicate miRNA overexpression, while downward red arrows represent downregulation. Created in https://BioRender.com (accessed on 23 January 2025).

PC3 and DU145 cell lines are widely used models in PCa research due to their metastatic origins and distinctive characteristics and differences in cell cycle regulation and DNA repair mechanisms, and metabolic profiles between the two cell lines could contribute to their varying sensitivities to treatments [43]. They have been widely utilized in various studies to explore the role of miRNAs, particularly their secretion via extracellular vesicles (EVs) derived from plasma, as prognostic biomarkers in CRPC. For instance, miRNAs have been assessed for their effects on cell proliferation and migration [44]. Additionally, research examining the impact of ionizing radiation on exosome secretion and cellular responses demonstrated that irradiated PC3 cells secrete significantly higher quantities of exosomes compared to DU145 cells, underscoring the role of exosomes in cancer progression and their potential as liquid biopsy markers [45]. Furthermore, investigations into TRAIL resistance in PCa cells cultured as 2D monolayers and 3D spheroids employed DU145 and PC3 cell lines to compare the sensitization of TRAIL-resistant cells through dimension-based chemotherapy [46]. In this study, we aim to investigate the dual role of miRNAs as potential serum biomarkers for distinguishing between ADT, sensitivity (responders), and resistance (non-responders) in PCa patients. Additionally, we utilize 3D culture models, specifically those derived from PC3 and DU145 cell lines, along with these cell lines-derived organoids, to explore the molecular mechanisms driving resistance and to assess innovative therapeutic strategies for addressing this complex and challenging disease (Figure 1).

## 2. Materials and Methods

### 2.1. Expression Profiles Analysis of miRNAs and Regulated mRNAs

Expression data of 99 and 203 differentially expressed miRNAs in CRPC were extracted from the dbDEMC database (https://www.biosino.org/dbDEMC (accessed on 18 September 2024)) with experiment ID number EXP00639 and EXP00640. Serum miRNAs of responder versus non-responder metastatic castration-resistant PCa patients to abiraterone acetate were extracted from GSE262550, and mRNA expression data from 3D of PC-3 cells were downloaded from GSE53245 from GEO Omnibus.

### 2.2. miRNAs Target Prediction and GSEA

miRNAs were filtered with fold change (1.5) and 0.05 *p*-value using the Grep function in R studio to filter the common miRNAs in datasets EXP00639, EXP00640, and GSE262550, obtaining 18 associated miRNAs after the miRNET https://www.mirnet.ca/ (accessed on 8 June 2024) and ENCORI database https://rnasysu.com/encori/panCancer.php (accessed on 9 June 2024) were use to determine mRNAs targets of miRNAs to analyze with Enricher database https://ethz-ins.org/enrichMiR/ (accessed 1 July 2024).

The following gene sets were downloaded from GSEA: the top 50 genes upregulated in metastatic versus primary PCa tumors and the androgen response hallmark. Subsequently, mRNAs upregulated in the GSE53245 dataset that were common with GSEA signatures were filtered according to miRNA prediction targets. Co-regulatory networks were then visualized using Cytoscape software https://cytoscape.org/ Version 3.10.3 (accessed 18 June 2024) [47].

### 2.3. TCGA-PRAD and ROC Plotter

TCGA-PRAD data from patients with PCa by Gleason score were used to determine the expression of miRNAs, analyzed using the UCSC Xena Browser database https://xenabrowser.net/ (accessed on 15 June 2024). Expression miRNAs of responder and non-responder samples and ROC curves were determined by the ROC-Plotter database https://ro|cplot.org/ (accessed on 20 June 2024).

### 2.4. Liquid Biopsies from Responders, Non-Responders PCa Patients, and Controls

Blood samples for liquid biopsy were obtained from PCa patients classified as responders (n = 20) and non-responders (n = 20), totaling 40 cases, along with a CTRL group of 20 individuals (n = 20). Participants were recruited at Hospital Juárez de México (HJM) based on the following criteria: males aged 40 years or older with a confirmed PCa diagnosis, undergoing ADT, presenting PSA levels above 4 ng/mL, and a Gleason score of at least 6. Exclusion criteria included men younger than 40, those with a family history of PCa, PSA levels below 4 ng/mL, or a prior diagnosis of another cancer type or severe disease. This work received ethical approval from the research and ethics committees of HJM (protocol HJM 009/23-I). After obtaining informed consent, 4–6 mL of blood was drawn using BD Vacutainer^®^ serum tubes (Beckton-Dickinson, Franklin Lakes, NJ, USA). The CTRL group comprised healthy men over 40 years old with PSA levels under 4 ng/mL and no history of PCa, other cancers, or severe illnesses. Hemolyzed serum samples were excluded from both the case and control groups, and all specimens were stored at −80 °C for subsequent analyses.

### 2.5. 3D Cell Lines Cultures from PC3 and DU145

PC3 and DU145 cell lines were obtained from ATCC (ATCC, Manassas, VA, USA) and cultured in F12 and low-glucose DMEM medium with 10% fetal bovine serum (FBS) (Gibco, Grand Island, NY, USA). To establish three-dimensional (3D) cultures, 120 µL of Matrigel (Thermo Fisher Scientific, Waltham, MA, USA) was added to 24-well plates and incubated at 37 °C for at least 30 min for matrix solidification. Subsequently, 250 µL of Hybri-Care medium (HCM)(ATCC) was added to 3.1 × 10^4^ cells from 2D PC3 and DU145 cultures and incubated at 37 °C for 30 min. Then, 250 µL of HCM enriched with 5% Matrigel (Thermo Fisher Scientific) was added and monitored for three days thereafter; the 3D cultures received 500 µL of fresh complete medium.

### 2.6. RNA Isolation from PC3 and DU145 Cells in 2D Monolayer and 3D Cultures

To analyze miRNA expression, total RNA was extracted from PC3 and DU145 cells cultured as 2D monolayers and 3D spheroids after six days of growth. The initial seeding density was 3.0 × 10^4^ cells, yielding approximately 62 ± 2.43 spheroids in the 3D system. For the extraction, 1 mL of TRIzol reagent (Thermo Fisher Scientific) was added to each culture type. The lysate was transferred to a 1.5 mL microcentrifuge tube and incubated at room temperature for 5 min. A total of 200 µL of chloroform was added per milliliter of TRIzol, and the mixture was shaken vigorously for 15 s, followed by incubation at room temperature for 3 min. The samples were centrifuged at 12,500 rpm for 25 min at 4 °C. The RNA aqueous phase was carefully transferred to a new tube, and RNA was precipitated by adding 500 µL of isopropanol. The samples were placed on ice for 20 min and then centrifuged at 12,500 rpm for another 25 min at 4 °C. After discarding the supernatant, the RNA pellet was washed with 1 mL of 75% ethanol, allowed to air-dry for 5 min, and resuspended in 20 µL of nuclease-free water. RNA concentration and purity were measured using a NanoDrop ND 2000 UV spectrophotometer (Thermo Fisher Scientific). The integrity of the RNA was verified through 1% agarose gel electrophoresis with 1× TAE buffer (Promega, Madison, WI, USA).

### 2.7. Reverse Transcription and Quantitative Real-Time PCR

The RNA extraction from serum was performed using TRIzol (Thermo Fisher Scientific). The extraction process followed the RNA extraction protocol provided by Invitrogen. cDNA was synthesized using the iScript™ cDNA Synthesis Kit (Bio-Rad). qRT-PCR assays were conducted with SYBR™ Green Universal Master Mix (Thermo Scientific) in a final volume of 10 µL. The primers used to detect to validate miRNAs in this study are listed in Table 1. The sequences of miRNA-specific primers for miR-341-3p, hsa-miR-411-5p, hsa-miR-629-3p, and hsa-miR-152-3p were obtained from the miRBase database https://www.mirbase.org/ (accessed on 21 June 2024). Primer design was performed using the miRprimer tool (accessed on 22 June 2024) https://tools4mirs.org/software/other_tools/mirprimer/), which enables the automatic design of primers for miR-specific RT-qPCR and U6 small nuclear RNA (RNU6) (forward primer 5′-CTCGCTTCGGCAGCACA.3′ and reverse primer 5′-AACGCTTCACGAATTTGCGT-3. All qPCRs were performed on the Bio-Rad CFX96 real-time PCR system. Data analysis was performed by the 2^−ΔΔCT^ method, where ΔCT = CT (cDNA1) − CT(cDNA2). In our case, CT (cDNA1) corresponds to the average CT normalized to the expression of the small nuclear RNU6 gene (snRNU6). The calculation of 2^−ΔΔCT^ yields normalized expression data that can be statistically compared subsequently.

### 2.8. Statistics Analysis

All data analyses were conducted using GraphPad Prism 8 www.graphpad.com (Windows version 8.2.1, San Diego, CA, USA). The results were expressed as mean ± SD and Student’s *t*-test, one-way or two-way ANOVA. To compare measurement data between groups a post hoc (Tukey) test was applied. A *p*-value < 0.001 was considered statistically significant.

## 3. Results

### 3.1. Comparative miRNA Analysis Unveiling Pathways of Resistance in Metastatic Castration-Resistant Prostate Cancer

A comprehensive comparative analysis of miRNAs was performed using multiple public datasets to identify key candidates associated with resistance mechanisms in metastatic CRPC. The first two datasets analyzed miRNA expression profiles from tissue samples of non-responder PCa patients who developed CRPC, while the third dataset evaluated miRNA expression in liquid biopsies (blood samples) from responders and non-responders to abiraterone acetate in metastatic settings. The primary objective was to identify miRNAs potentially involved in resistance-related signaling pathways and detectable in liquid biopsies.

**Figure 2:**
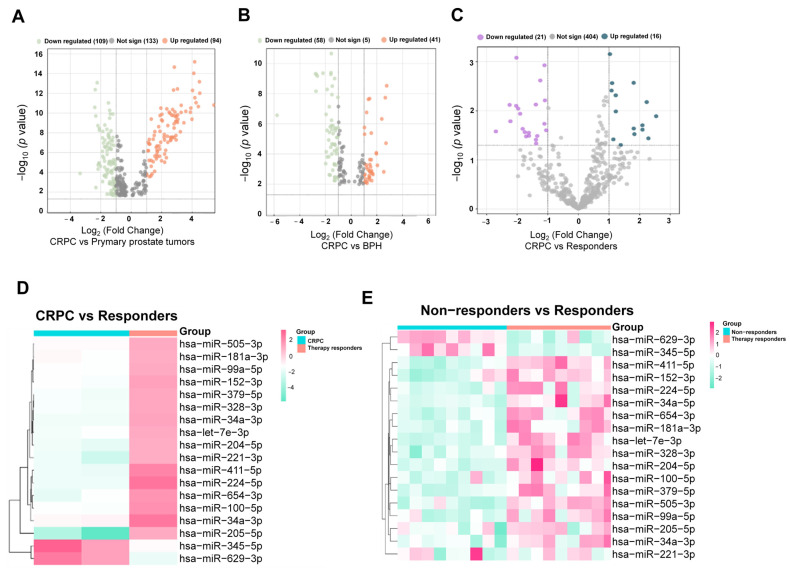
miRNA expression profiles in non-responders CRPC datasets. (**A**,**B**) Volcano plot datasets of miRNAs with differential expression in CRPC vs. primary prostate tumors and BPH (fold change > 1.5 and *p*-value 0.05). (**C**) Volcano plot dataset of miRNAs plasma miRNAs of responder versus non-responder metastatic castration-resistant PCa patients to abiraterone acetate (fold change > 1.5, *p*-value of 0.05). (**D**) Common miRNAs across the three datasets. (**E**) Heatmap of common miRNAs in non-responders vs. responders (average linkage based on Euclidean distance measurements). Aqua blue color represents downregulation, and pink represents upregulation.

In tissue samples, the datasets revealed the differential expression of 94 and 41 upregulated miRNAs and 109 and 58 downregulated miRNAs, respectively (Figure 2A,B). In liquid biopsies, 16 miRNAs were found to be upregulated and 21 downregulated in non-responders compared to responders (Figure 2C). A fold change threshold of 1.5 and a *p*-value of 0.05 were applied as cut-off criteria for all datasets. Across the three datasets, 18 common miRNAs were identified. Among these, 16 miRNAs were overexpressed in responders to abiraterone but repressed in non-responder samples, whereas two miRNAs exhibited the opposite pattern, being downregulated in responders but upregulated in non-responders (Figure 2D).

To illustrate the expression patterns of these miRNAs, heatmaps were generated. Distinct clusters of expression were observed, highlighting miRNAs such as hsa-miR-345-5p, hsa-miR-629-3p, hsa-miR-152-3p, hsa-miR-654-3p, hsa-miR-411-5p, and hsa-miR-34a-3p as particularly notable candidates (Figure 2E). These findings underscore the potential of these miRNAs to act as biomarkers for therapeutic response and as targets for further investigation into resistance mechanisms in CRPC.

### 3.2. Differentially Expressed miRNAs hsa-miR-152-3p, hsa-miR-654-3p, hsa-miR-411-5p, and hsa-miR-34a-3p Are Associated with Metastasis and Androgen Response

**Figure 3:**
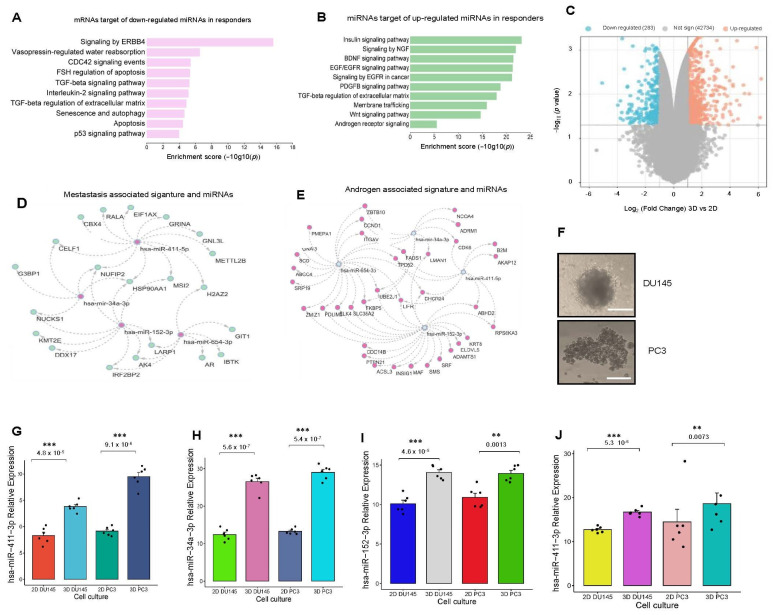
miRNA expression profiles and targets in responder patients and identified pathways associated. (**A**) Pathways and biological processes related to miRNAs downregulated in responder patients. (**B**) Pathways and biological processes related to upregulated miRNAs in responder PCa patients. (**C**) Volcano plot datasets of mRNAs with differential expression in 3D culture vs. 2D culture from PC3 cells (fold change > 1.5, *p*-value 0.05). (**D**) miRNA network regulation of top 50 genes upregulated in metastatic vs. primary PCa tumors from GSEA signature. (**E**) miRNA network regulation of top 50 genes upregulated in metastatic vs. primary PCa tumors from GSEA signature. (**F**) Representative 3D cell cultures of DU145 and PC3 cells observed by optical microscopy (40×) after 6 days of incubation over Matrigel. Bar represents 150 nm. Relative hsa-miR-411-3p (**G**), miR-34a-p (**H**), miR-152-3p (**I**), and miR-654a-3p (**J**) expression in 2D and 3D cell lines cultures of DU145 and PC3, respectively. The relative levels of miRNAs were obtained by qRT-PCR and calculated using 2^−ΔΔCt^. The box-plot graph indicates the median with quartiles, and error bars represent ± SD (two independent experiments by triplicate for each cell culture condition). A *t*-test and Tukey test were used for comparison; *** indicates *p* < 0.001, ** indicates *p* < 0.01. snRNU6 expression levels were used for the normalization of data.

To investigate the roles of specific miRNAs in PCa progression, we performed a target prediction analysis to identify their associated signaling pathways and biological processes. The analysis categorized processes according to expression changes of miRNAs. In patients responding to abiraterone treatment, repressed miRNAs targeted mRNAs involved in pathways such as receptor tyrosine-protein kinase erbB-4 (ERBB4), transforming growth factor β (TGF-β), p53 signaling, and cellular processes like senescence and apoptosis (Figure 3A). Conversely, miRNAs overexpressed in responders were found to inhibit pathways related to insulin signaling, nerve growth factor (NGF), epidermal growth factor receptor (EGFR), WNT signaling, and AR signaling. These processes are closely linked to the progression from androgen dependence to castration resistance (Figure 3B). A deeper exploration was conducted using another public dataset of differentially expressed mRNAs from three-dimensional (3D) cultures of the PC3 PCa cell line, which is known for its high aggressiveness and metastatic potential, making it a relevant model for castration-resistant tumors [9] (Figure 2C).

Among 565 mRNAs overexpressed in the 3D cultures (fold change > 1.5, *p*-value < 0.05), their involvement in two Gene Set Enrichment Analysis (GSEA) signature sets was examined: genes upregulated in metastatic versus primary PCa tumors and the androgen response hallmark. This analysis demonstrated that miRNAs such as hsa-miR-152-3p, hsa-miR-34a-3p, hsa-miR-654-3p, and hsa-miR-411-5p play regulatory roles in suppressing genes associated with metastasis (Figure 3D) and androgen response (Figure 3E).

To validate these findings, miRNA expression profiles were analyzed in both 2D and 3D cultures of PC3 and DU145 cell lines—two of the most widely used models for studying androgen-dependent and castration-resistant models in PCa. In 3D cultures, DU145 cells, an AR mutated non-hormone sensitive and negative PSA PCa cell line with a moderate metastatic potential, formed spheroid-like structures by day 6 on Matrigel, while PC3 cells displayed a “collapsed” structure, consistent with prior reports (Figure 3F,G) [3]. These structural differences may reflect the inherent characteristics of the cell lines; DU145 cells exhibit features of adenocarcinoma, whereas PC3 cells exhibit epithelial morphology, non-hormone sensitive, and negative PSA cell line that typically represent prostatic small cell neuroendocrine carcinoma (SCNC), a more aggressive PCa subtype. Despite these phenotypic differences, both cell lines effectively model distinct aspects of PCa biology [9].

These analyses confirmed a robust correlation between miRNA expression levels observed in public datasets and those detected in 3D cultures. Specifically, hsa-miR-411-5p, hsa-miR-34a-3p, hsa-miR-152-3p, and hsa-miR-654a-3p were significantly overexpressed in 3D cultures of PC3 and DU145 cells compared to their respective 2D monolayer cultures (Figure 3F–J). These results also highlight the importance of 3D culture systems in understanding miRNA roles in the tumor microenvironment and their potential roles as therapeutic targets in PCa.

### 3.3. Expression Profiles of Therapy Response miRNAs Are Inversely Associated with Therapy Response Data

Studies have shown that miRNA expression profiles are strongly associated with therapy responses in PCa, particularly in cases of hormone resistance [48]. Using data from The Cancer Genome Atlas Prostate Adenocarcinoma (TCGA-PRAD), we evaluated the expression levels of specific miRNAs in relation to the Gleason score, a key prognostic marker for PCa [49].

**Figure 4:**
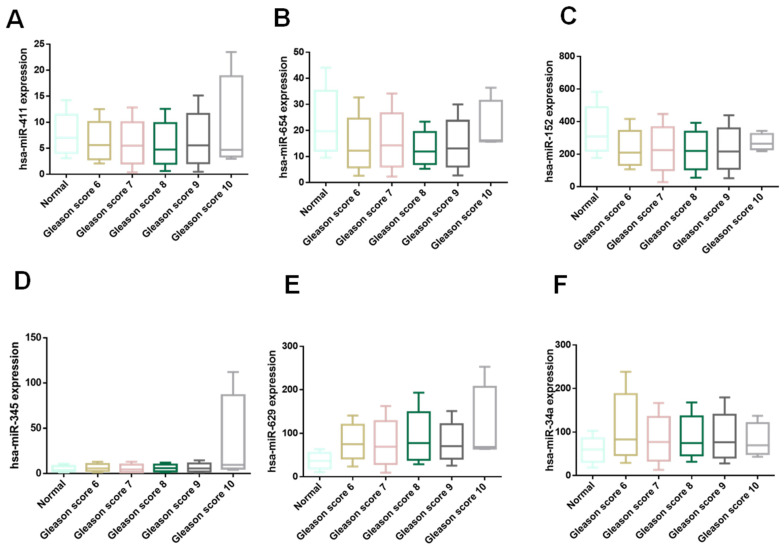
miRNAs expression profiles by Gleason score from PRAD-TGCA. Expression profile of miRNAa in patient tissue. Profile expression obtained from the TCGA-PRAD database through the Xena Browser comparing normal tissue and PCa tissue. (**A**) hsa-miR-411, (**B**) hsa-miR-654, (**C**) hsa-miR-152, (**D**) hsa-miR-345, (**E**) hsa-miR-629, and (**F**) hsa-miR-34a. Mean comparison was performed by ANOVA Tukey test. A fold change of 1.5 and a *p*-value of <0.05 were used as cut-off values.

Our analysis revealed distinct expression patterns for various miRNAs. For instance, hsa-miR-411 was significantly overexpressed in normal tissues compared to tumor tissues at Gleason stages 7, 8, and 9 (Figure 4A). Similarly, hsa-miR-152-3p and hsa-miR-654-3p were markedly overexpressed in normal tissues compared to tumor tissues across Gleason stages 6-9 (Figure 4B,C). These miRNAs appear to exhibit tumor-suppressive characteristics, potentially playing protective roles in healthy tissues. In contrast, hsa-miR-345 was significantly overexpressed in tumor tissues at Gleason stages 6, 8, and 9 (Figure 4D), indicating its potential as an oncogenic miRNA. Likewise, hsa-miR-629 showed consistent overexpression across all Gleason stages (6–10) when compared to normal tissues (Figure 4E), suggesting its involvement in tumor progression. Interestingly, hsa-miR-34a displayed a discordant expression pattern, with elevated levels across all Gleason stages relative to normal tissues (Figure 4F), highlighting its complex regulatory role.

These findings align with observations from public datasets. Specifically, hsa-miR-152-3p, hsa-miR-654-3p, and hsa-miR-411 were consistently overexpressed in patients who responded to abiraterone therapy and in normal tissues compared to tumor tissues stratified by Gleason score. Notably, hsa-miR-629 and hsa-miR-411 also exhibited elevated expression in abiraterone-resistant castration states and in tumor tissues with high Gleason scores. This correlation emphasizes the potential role of these miRNAs as biomarkers for predicting therapy response and stratifying patients based on disease aggressiveness.

### 3.4. Expression Profile of hsa-miR-152-3p, hsa-miR-411-5p, and hsa-miR-34a-3p as Potential Predictors of Therapy Response

To explore the predictive potential of specific miRNAs in therapy response for PCa, we employed the ROC Plotter database to evaluate the expression profiles of six candidate miRNAs: hsa-miR-345-5p, hsa-miR-629-3p, hsa-miR-152-3p, hsa-miR-654-3p, hsa-miR-411-5p, and hsa-miR-34a-3p. By correlating miRNA expression levels with clinical outcomes in cancer patients, this analysis aimed to identify biomarkers with the capacity to stratify patients based on their therapeutic response.

**Figure 5:**
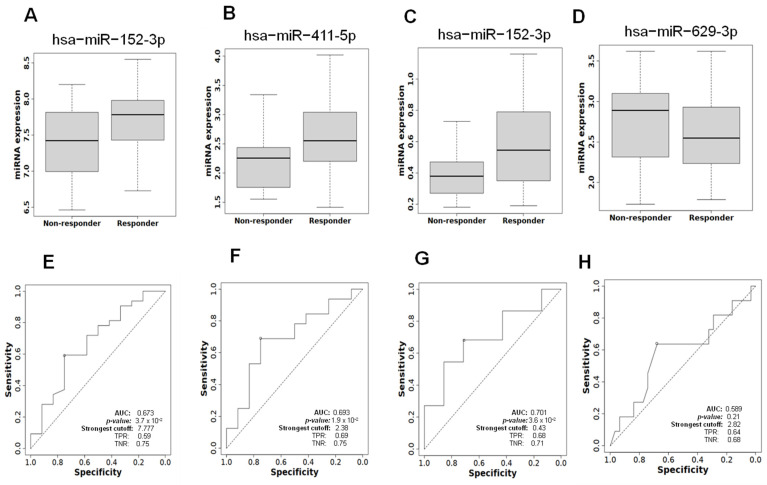
miRNA expression profiles and correlation with Gleason score progression and aggressiveness from PRAD-TCGA. Comparison of expression miRNA levels of responder and non-responder PCa patients of (**A**) has-miR-152-3p, (**B**) has-miR-411-5p, (**C**) has-miR-34a-3p, and (**D**) hsa-miR-629-3p. The length of the bars represents the miRNA expression levels. Error bars represent ± SD, and mean comparison using a *t*-test was used to generate the *p*-values with a significance threshold set at 0.05. Evaluation of ROC curves and AUC in responder and non-responder patients. ROC and AUC values from (**E**) hsa-miR-152-3p, (**F**) hsa-miR-411-5p, (**G**) hsa-miR-34a-5p, and (**H**) hsa-miR-629-3p were calculated to assess the feasibility of using miRNA levels from patients as a potential diagnostic tool, highlighting significant differences that may correlate with PCa.

The results highlighted three miRNAs—hsa-miR-152-3p, hsa-miR-411-5p, and hsa-miR-34a-3p—as promising candidates for predicting therapy response (Figure 5A–D). These miRNAs were notably overexpressed in patients who responded positively to treatment. Importantly, their area under the curve (AUC) values ranged from 0.67 to 0.70, suggesting moderate discriminative ability. An AUC of 0.70, for instance, indicates that the corresponding miRNA has a 70% probability of accurately distinguishing between therapy responders and non-responders (Figure 5E–H).

The identification of these miRNAs as predictive biomarkers holds significant clinical relevance. Reliable biomarkers for therapy response can enable more precise, personalized treatment strategies, potentially improving patient outcomes by optimizing therapeutic efficacy and minimizing unnecessary interventions. While these results are promising, further validation in larger and ethnically diverse patient cohorts is essential to solidify the utility of these miRNAs in clinical outcomes.

### 3.5. Validation of miRNA Expression Related to Therapy Response in a Cohort of Mexican Prostate Cancer Patients

Finally, we evaluated the expression levels of key miRNAs in a cohort of Mexican PCa patients to determine their potential as biomarkers for therapy response. A total of 20 blood samples from non-responders (Supplementary Table S1) and 20 samples from responders to therapy were analyzed, with healthy volunteers serving as CTRL. The miRNAs examined included hsa-miR-34a-3p, hsa-miR-411-5p, hsa-miR-629-3p, and hsa-miR-152-3p.

**Figure 6:**
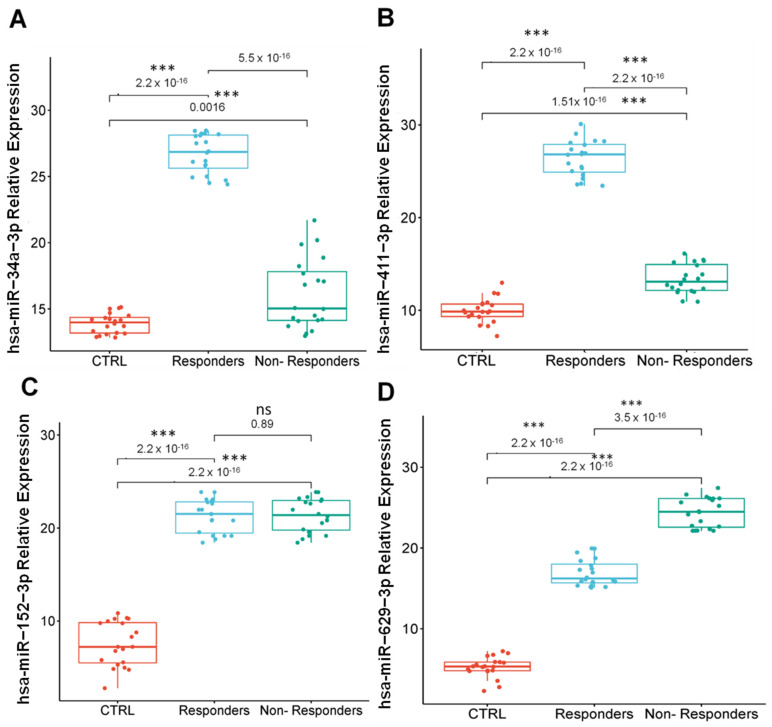
Relative expression of selected miRNAs (**A**) has-miR-34a-3p, (**B**) has-miR-152-3p, (**C**) hsa-miR-411-5p, and (**D**) hsa-miR-629-3p in serum from responder (NCRPC) and non-responder (CRPC) PCa patients. miRNAs relative expression levels were determined by qRT-PCR and calculated with 2^−ΔΔCt^. The box-plot graph shows the median with quartile. ±SD are represented by error bars (two independent experiments by technical triplicate). Mean comparison using ANOVA test. ***: *p* < 0.001. ns: not statically significance. The normalization of miRNA levels was performed with snRNU6.

The analysis revealed consistent expression patterns between the Mexican cohort and previously reported public datasets. Specifically, hsa-miR-34a-3p and hsa-miR-411-5p were significantly overexpressed in responders (Figure 6A,B), whereas hsa-miR-629-3p was predominantly overexpressed in non-responders (Figure 6C). However, contrary to prior findings from TCGA-PRAD data, hsa-miR-152-3p exhibited no significant differences between responders and non-responders in this cohort (Figure 6D). While hsa-miR-152-3p was reported overexpressed in responders, its uniform expression in this study underscores the necessity of validating miRNA biomarkers in diverse populations to establish their universal applicability.

The serum miRNA expression profiles observed align with prior studies implicating these miRNAs in cancer progression and resistance mechanisms. For example, hsa-miR-34a-3p has demonstrated tumor-suppressive effects in PCa, including inhibition of cell proliferation and cell cycle arrest induction [50,51,52,53]. Similarly, hsa-miR-411-5p, which was markedly overexpressed in responders, is known to inhibit PCa progression both in vitro and in vivo [54], which suggests a potential role in modulating therapeutic outcomes. Additionally, hsa-miR-152-3p, which showed similar expression levels in both groups in our study, is recognized for its tumor-suppressive roles [55]. Conversely, hsa-miR-629-3p was upregulated in non-responders, consistent with its established oncogenic role and promotion of cell proliferation, possibly implicating it in therapy resistance [56,57].

These findings reinforce the importance of miRNAs as biomarkers in predicting therapy response, providing a strong foundation for future research. Investigations to elucidate the mechanistic roles of these miRNAs, their molecular targets, and their interactions with signaling pathways are essential to fully understand their potential as therapeutic targets. Additionally, these results highlight the need for population-specific validation studies to ensure the reliability and clinical utility of miRNA biomarkers across diverse patient groups.

## 4. Discussion

miRNAs are strongly linked to the aggressiveness and health disparities seen in PCa, potentially aiding in the prediction of prognosis and the development of treatment strategies. Moreover, miRNA expression and regulation may serve as risk factors for prognosis and offer preventative and therapeutic options for men at higher risk of PCa, particularly within specific populations [58]. Recent studies have also indicated that differential expression of miRNA is associated with the aggressiveness of PCa and may provide more precise prognostic tools, specifically for patients with CRPC [59].

In addition, miRNAs appear to play a key role in the interaction between the AR and non-responder patients to therapy, also known as CRPC. Research has shown that changes in miRNA expression during the progression and persistence of CRPC may be associated with the upregulation of certain miRNAs [60]. For instance, miRNAs such as miR-21 and miR-375 have been linked to increased resistance to hormonal therapy [61], suggesting their involvement in AR regulation. Liquid biopsy biomarkers like circulating tumor DNA (ctDNA), circulating tumor cells (CTCs), and extracellular vesicles (EVs) containing miRNAs are a transformative approach to PCa diagnostics and prognostics, offering the potential to refine pre-biopsy decision-making and differentiate between patients requiring immediate treatment and those suitable for active surveillance [62].

Our study revealed distinct expression profiles of specific miRNAs in relation to therapy response in PCa patients. For example, hsa-miR-34a-3p and hsa-miR-411-5p were statically increased in the serum of patients who responded to therapy. These miRNAs are also highly expressed in normal tissues but repressed in tumor tissues, as demonstrated by TCGA-PRAD data. Importantly, these miRNAs target genes associated with metastasis as NUCKS1, mainly in non-small cell lung and breast cancer [63,64], and androgen response, G3BP1 [65], suggesting their potential role in suppressing the progression of tumors and enhancing therapy sensitivity. The TCGA-PRAD data further corroborate these findings, showing that these miRNAs are overexpressed in tumor tissues and in samples from CRPC patients. The inverse relationship between miRNA expression and therapy response indicates their involvement in promoting resistance mechanisms and tumor aggressiveness [66].

Our findings also highlight the potential of these miRNAs as biomarkers for predicting therapy response and understanding the molecular underpinnings of PCa progression. The differential expression patterns suggest that hsa-miR-34a-3p, hsa-miR-411-5p, hsa-miR-152-3p, and hsa-miR-654-3p may function as tumor suppressors, while hsa-miR-345-5p and hsa-miR-629-3p might act as oncogenic miRNAs. miRNA profiles can reliably predict malignancies, and these unique expression patterns, which have been identified across various tumor types, may serve as specific phenotypic markers for cancer detection, prognosis, and treatment strategies [67]. This positions miRNAs as powerful tools for addressing many of the current challenges in cancer diagnostics, including metastasis in PCa [68].

miRNAs have dual functions as tumor suppressors or act as oncogenes in the context, depending on the genes they target, with implications in the transformation of cancer cells. For example, oncomiRs, a specific class of miRNAs, exhibit elevated expression levels in tumors, where they suppress tumor-suppressor mRNAs and promote tumor growth and metastasis [69,70]. These miRNAs play diverse roles in malign progression, contributing significantly to the regulation of cellular mechanisms that drive tumor development and spread. Previous studies have found that miR-411-5p is downregulated in PCa cells, and its restoration can inhibit the migration and invasion of CWR22RV1 and LNCaP cells [54]. Additionally, miR-34a-3p is known to be a p53 transcriptional target that is significantly downregulated in PCa with sustained p53 loss or mutations [71] and the restoration of this miRNA represses CD44 inhibiting PCa stem cells phenotype and metastasis [72]. miR-34a-3p has also been identified as having tumor-suppressive effects on PCa by inhibiting the migration and subsequent proliferation of PC3 cells, arresting the cell cycle in the G2 phase and targeting the Wnt signaling pathway [50,51,52,53].

Regarding miR-152-3p, we encountered some controversies. Some authors report that this miRNA is upregulated in plasma from PCa patients compared to sample CTRL [73] and has been shown to promote PCa metastasis by suppressing PTEN. However, other studies suggest that miR-152-3p is underexpressed in PCa, associated with promoter hypermethylation, according to TCGA dataset analysis. In vitro assays indicate suppressed cell viability and invasion potential by miR-152-3p and promote cell cycle arrest at the S and G2/M phases [55]. We found no significant differences in the miR-152-3p expression profile between responder and non-responder patients; therefore, it cannot be ruled out that it may still perform these described functions in PCa. It should also be considered that our cohort consists of a specific group of patients who have received ADT and have developed, or not developed, resistance.

Reports related to miR-654-3p show that it regulates the malignant phenotype and angiogenesis of PCa cells, associated with the sponge effect of circRNA circ_0001165 [74]. Conversely, hsa-miR-629 was overexpressed in non-responders, suggesting a function as a marker of therapy resistance [75,76]. However, its discriminative power, as indicated by its AUC value, was not significant. This underscores the complexity of miRNA regulation in PCa and highlights the need for further research to elucidate the mechanisms underlying therapy resistance. miRNA levels might serve as valuable biomarkers for diagnosis and identifying aggressiveness or micrometastases in patients and predicting potential ADT recurrence. However, larger cohort studies are essential to validate these findings before they can be translated into clinical practice. Such studies would enhance diagnostic precision and pave the way for personalized treatment strategies tailored to PCa patients. Finally, miR-629-5p levels are increased in primary localized and metastatic PCa compared with adjacent normal tissues. Its expression is associated with poor prognosis and, in vitro, drives cell proliferation, migration, and invasion but promotes tumor growth in vivo [57].

Tumor development is related to most studied miRNAs that play important roles in cancer progression and, as demonstrated here, are promising for PCa biomarkers and treatment. In this study, we focus on the potential circulating biomarker function of these miRNAs in the TCGA-PRAD dataset to evaluate their potential accuracy in detecting clinical stages of PCa progression, especially in therapy non-responders classified as CRPC patients. However, one limitation of this study lies in its relatively small sample size and population. To confirm these preliminary findings and establish the clinical utility of these miRNAs, larger cohort and multi-ethnic cohort studies are essential to ensure the robustness and generalizability of these potential biomarkers. Although this study is centered on a Mexican cohort, future research should explore its broader applicability to diverse populations to ensure the generalizability and translational value of the findings in varied clinical and genetic contexts [77]. Although the findings are consistent, validation in larger cohorts would be essential to ensure the reliability of results [78,79]. Such confirmation is necessary before considering the translational application of these insights in clinical settings to enhance diagnostic precision and guide personalized treatment strategies for PCa patients. The integration of miRNAs with other liquid biopsy biomarkers, including ctDNA and protein markers, has the potential to significantly improve diagnostic accuracy and facilitate personalized treatment strategies by providing a comprehensive perspective of the tumor microenvironment [80,81].

Additionally, investigating the biological functions and target pathways of these miRNAs could provide insights into their roles in tumor biology and therapy response, demonstrating the importance of complementing these studies with patient-derived xenografts or organoid models. Several miRNAs have multiple cellular processes and important pathways that implicate cell survival, such as ER stress, necrosis, autophagy, and cancer progression. Furthermore, experimental validation is essential to elucidate the molecular mechanisms through which specific miRNAs may contribute to the development and progression of CRPC. Future research should also explore the integration of miRNA profiling with other molecular and clinical parameters to enhance predictive accuracy and develop comprehensive models for patient stratification in PCa. This will help elucidate the precise roles of these miRNAs in PCa and their potential utility in clinical practice.

## Figures and Tables

**Table 1:** Primer sequences of miRNAs analyzed in this study.

miRNA	Sequence Target	Primer Sequence
hsa-miR-34a-3p	CAAUCAGCAAGUAUACUGCCCU (https://www.mirbase.org/mature/MIMAT0004557) (accessed on 21 June 2024).	F: 5′-CGCAGCAATCAGCAAGT-3′ R: 5′-CAGTTTTTTTTTTTTTTTAGGGCAGT-3′
hsa-miR-411-5p	UAGUAGACCGUAUAGCGUACG (https://www.mirbase.org/mature/MIMAT0003329) (accessed on 21 June 2024).	F: 5′-CAGTAGTAGACCGTATAGCGT-3′ R: 5′-GGTCCAGTTTTTTTTTTTTTTTCGT-3′
hsa-miR-629-3p	GUUCUCCCAACGUAAGCCCAGC (https://www.mirbase.org/mature/MIMAT0003298) (accessed on 21 June 2024).	F: 5′-CAGGTTCTCCCAACGTAAG-3′ R: 5′-GTCCAGTTTTTTTTTTTTTTTGCTG 3′
hsa-miR-152-3p	UCAGUGCAUGACAGAACUUGG (https://www.mirbase.org/mature/MIMAT0000438) (accessed on 21 June 2024).	F: 5′-GCAGTCAGTGCATGACAGA-3′ R: 5′-GTCCAGTTTTTTTTTTTTTTTCCAAG-3′

F: Forward oligonucleotide. R: reverse oligonucleotide.

## Data Availability

The original data presented in the study are openly available at [https://portal.gdc.cancer.gov/projects/TCGA-PRAD], and GEO omnibus datasets GSE262550 and GSE53245 are available at [https://www.ncbi.nlm.nih.gov/geo/], accessed on 5 September 2024.

## Supplementary Materials

The following supporting information can be downloaded at https://www.mdpi.com/article/10.3390/genes16020180/s1, Table S1: Clinical stage Gleason score of non-responder PCa cases.

## Abbreviations

The following abbreviations are used in this manuscript:

PCa	Prostate cancer
CRPC	Castration-resistant prostate cancer
ADT	Androgen deprivation therapy
miRNAs	MicroRNAs
ROC	Receiver Operating Characteristic
PSA	Prostatic Specific Antigen
FBS	Fetal Bovine Serum
HCM	Hybri-Care medium
ERBB4	Receptor tyrosine-protein kinase erbB-4
TGF-β	Transforming growth factor β
NGF	Nerve growth factor
EGFR	Epidermal growth factor receptor
AR	Androgen receptor
