# Supplementary material for: miRNA Signatures as Predictors of Therapy Response in Castration-Resistant Prostate Cancer: Insights from Clinical Liquid Biopsies and 3D Culture Models
Source: Genes (Basel). 2025 Feb 1;16(2):180. doi: 10.3390/genes16020180 (PMC11855684; doi:10.3390/genes16020180)
Supplement: Supplementary file 1 [file genes-16-00180-s001.zip › genes-3445551-supplementary.pdf]

Supplementary Table S1. Clinical stage Gleason score of non-responders PCa cases

Clinical Stage	CS cases (n)		
		**Cases(n)**	**Gleason Score[1]**
CSI	3	1	6 (3+3)
		1	7 (4+3)
		1	8 (4+4)
CSII	3	1	7 (4+3)
		1	8 (4+4)
		1	9 (5+4)
CSIII	6	3	7 (4+3)
		2	6 (4+2)
		1	8 (4+4)
CSIV	8	4	9
		1	8
		1	10
		1	7
		1	6
